# Supplementary material for: A robust and scalable framework for hallucination detection in virtual tissue staining and digital pathology
Source: Nat Biomed Eng. 2025 Jun 16;9(12):2196–214. doi: 10.1038/s41551-025-01421-9 (PMC12705451; doi:10.1038/s41551-025-01421-9)
Supplement: Supplementary file 1 — Supplementary Notes 1–3 and Figs. 1–12. [file 41551_2025_1421_MOESM1_ESM.pdf]

nature biomedical engineering

Supplementary information

https://doi.org/10.1038/s41551-025-01421-9

A robust and scalable framework for hallucination detection in virtual tissue staining and digital pathology

In the format provided by the authors and unedited

Supplementary Information

Supplementary Note 1 Grad-CAM visualization analysis

In this analysis, we visualize the spatial-temporal distribution of attributing features in AQuA inference on poor VS images of human kidney samples. Leveraging the activation maps within the residual blocks of the ResNet-50 backbone in AQuA-Net and the gradient from the positive class label, heatmaps are generated, overlapped with the VS image and displayed for VS images at each time step VS(t); see the Methods section for implementation details. In addition, heatmaps are averaged over time, denoted as <Grad-CAM> to show the spatial locations of attributing features. For reference, the squared difference map between the VS image and HS reference image is also calculated and smoothed by a Gaussian kernel k to match the resolution of Grad-CAM. Extended Data Figure 10 illustrates VS-AF iterations of a poor VS image and the corresponding Grad-CAM heatmaps. AQuA correctly classifies this VS image as positive. As shown by Extended Data Fig. 10(c), heatmaps generated by AQuA without the HS reference image correlate well with the difference map calculated with respect to the HS reference image, confirming its autonomous quality assessment ability to serve as a watchdog in the VS workflow. Specifically, as pointed out by the arrows of Extended Data Fig. 10(d), a relatively large tissue area is erased by the poor-staining VS network model and replaced by the hallucinatory boundaries of the tissue. Without the HS reference image, AQuA successfully detects this abnormal and inconsistent hallucination and highlights this area in the heatmaps. AQuA's decision is also attributed to small areas that are rare or abnormal in common good-quality VS images, including areas with high AF background noise and residual tissue, as pointed out by Extended Data Fig. 10(e) and (f), respectively. In contrast, the difference map based on the HS reference image fails to detect such minor inconsistencies or abnormalities. Supplementary Figures 4-5 provide two additional examples of Grad-CAM visualization. In Supplementary Fig. 4, regions with severe artifacts pointed by (f, g) are captured by both the difference map and AQuA without relying on the HS reference image. Furthermore, Supplementary Figs. 4(d, e, h) highlight hallucinatory artifacts successfully detected by AQuA but not recognized by the difference map against HS reference. Supplementary Figure 5 shows another FOV of Grad-CAM visualization. Small fragmented, detached areas indicated by Supplementary Fig. 5(d, e) are successfully detected by AQuA. In the difference map against the HS reference image, these areas are also highlighted as they were possibly washed away during the staining process. Hallucinatory regions with abnormally high AF intensities indicated by Supplementary Fig. 5(f, g) are also successfully detected by AQuA, but the difference map failed to capture them as the hallucination is intricate and relatively minor in terms of the MSE. These observations confirm the difficulty of autonomous VS quality assessment task using traditional structural metrics-based terms and further emphasize the merits of the AQuA framework.

Supplementary Note 2 Analysis of nuclei count differences between VS and HS images, as well as between serially sectioned adjacent HS tissue cuts.

In traditional histopathological workflows, even adjacent tissue slides from the same sample of the same patient inevitably have intrinsic variance due to tissue heterogeneity. In diagnosing the potential lung transplant rejection, nuclei aggregation within a local region provides critical diagnostic information for the decision. However, the degree of change in terms of nuclei counts and aggregation observed between VS and the HS reference is even smaller than the variations observed between adjacent tissue sections of the same tissue block. For the two FOVs included in Extended Data Figure 4, a detailed comparison of nuclei count differences is provided in Supplementary Figure 2. This includes comparisons between HS and VS for each FOV, as well as between HS and its adjacent section cuts histochemically stained with different stain types (Masson's Trichrome (MT) stain and Elastic Verhoeff-Van Gieson (EVG) stain) for each FOV. These comparisons clearly demonstrate that the nuclei count discrepancies between HS and VS are smaller than those observed between HS and its adjacent section cuts.

Supplementary Note 3 Comparison between VS and HS quality

To validate the inference performance of the VS models used in the iterative inference, we conducted a quantitative study on kidney tissue samples to compare the similarity between VS H&E images and their HS counterparts. Supplementary Figure 10 presents comparisons of the color distributions of 76 paired VS and HS FOVs (1024×1024 pixels) in the YCbCr color space and separated eosin and hematoxylin staining channels, demonstrating a high level of overlap between the virtual and histochemical staining. Additionally, as summarized in Supplementary Figure 11, the VS H&E images and their HS equivalents were further evaluated and compared using standard metrics such as PSNR and SSIM, along with different features, including the number of nuclei per FOV and the average area of nuclei. The high average PSNR (23.8241) and SSIM (0.8817) values also confirmed the success of the virtual staining performance. Furthermore, the closely matching distributions for the number of nuclei per FOV and average area of nuclei, together with the calculated Hellinger distances and related statistical analyses, substantiate that there is no statistically significant difference between the VS images and their HS counterparts, further validating the reliability of the G_{VS} model used in the VS-AF iterations.

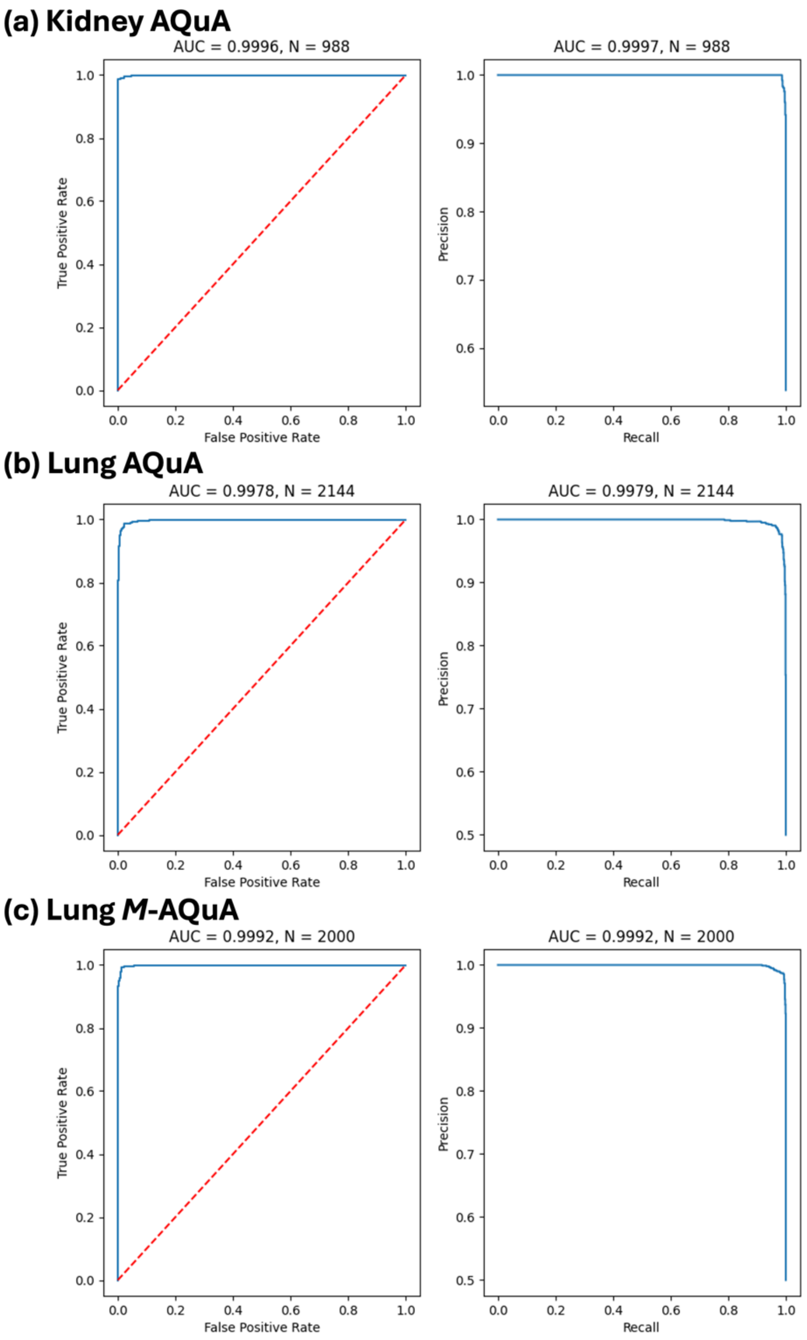

Supplementary Figure 1 Receiver operation characteristic (ROC) curve and precision-recall curve (PRC) of (a) kidney and (b) lung VS AQuA models. (c) ROC and PRC curves of *M*-AQuA model on lung VS images. AUC: area under the curve; N: the number of samples in the testing set. Metrics are calculated on testing sets of the corresponding organ type.

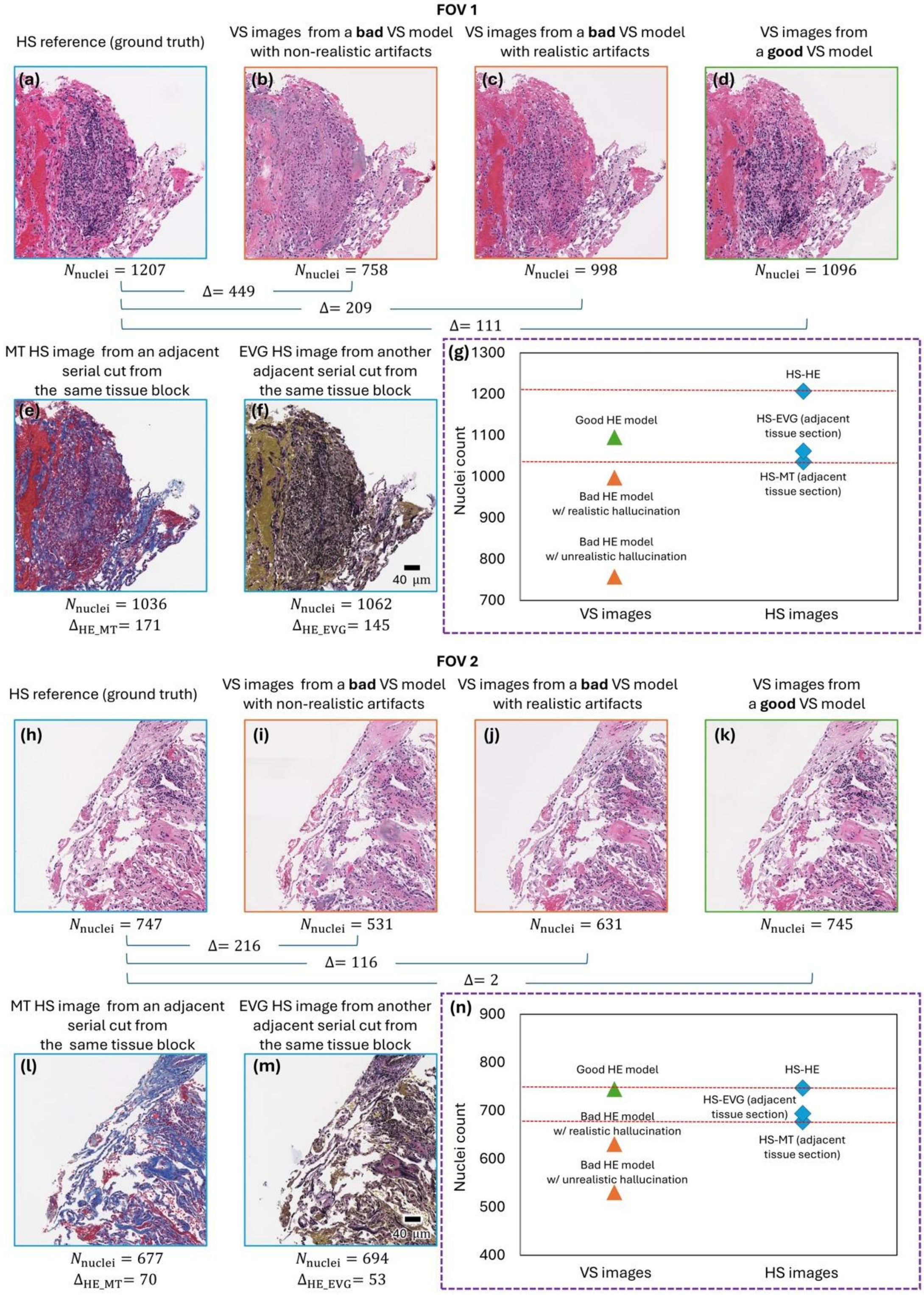

Supplementary Figure 2 Quantification of nuclei count differences between VS and HS images, as well as between serially sectioned adjacent HS tissue cuts stained with HE, MT, and EVG. (a) Reference HE-stained HS image. (b-d) VS images generated by a bad VS model with non-realistic hallucinations, a bad VS model with realistic hallucinations, and a good VS model, respectively. (e) HS image from an adjacent tissue section stained with MT. (f) HS image from an adjacent tissue section stained with EVG. (g) Plot of nuclei counts for images (a-f), demonstrating that the differences between good VS and HS images are comparatively smaller than the differences observed between HS and its adjacent tissue cuts. (h-n) Same as in (a-g) but for a second FOV.

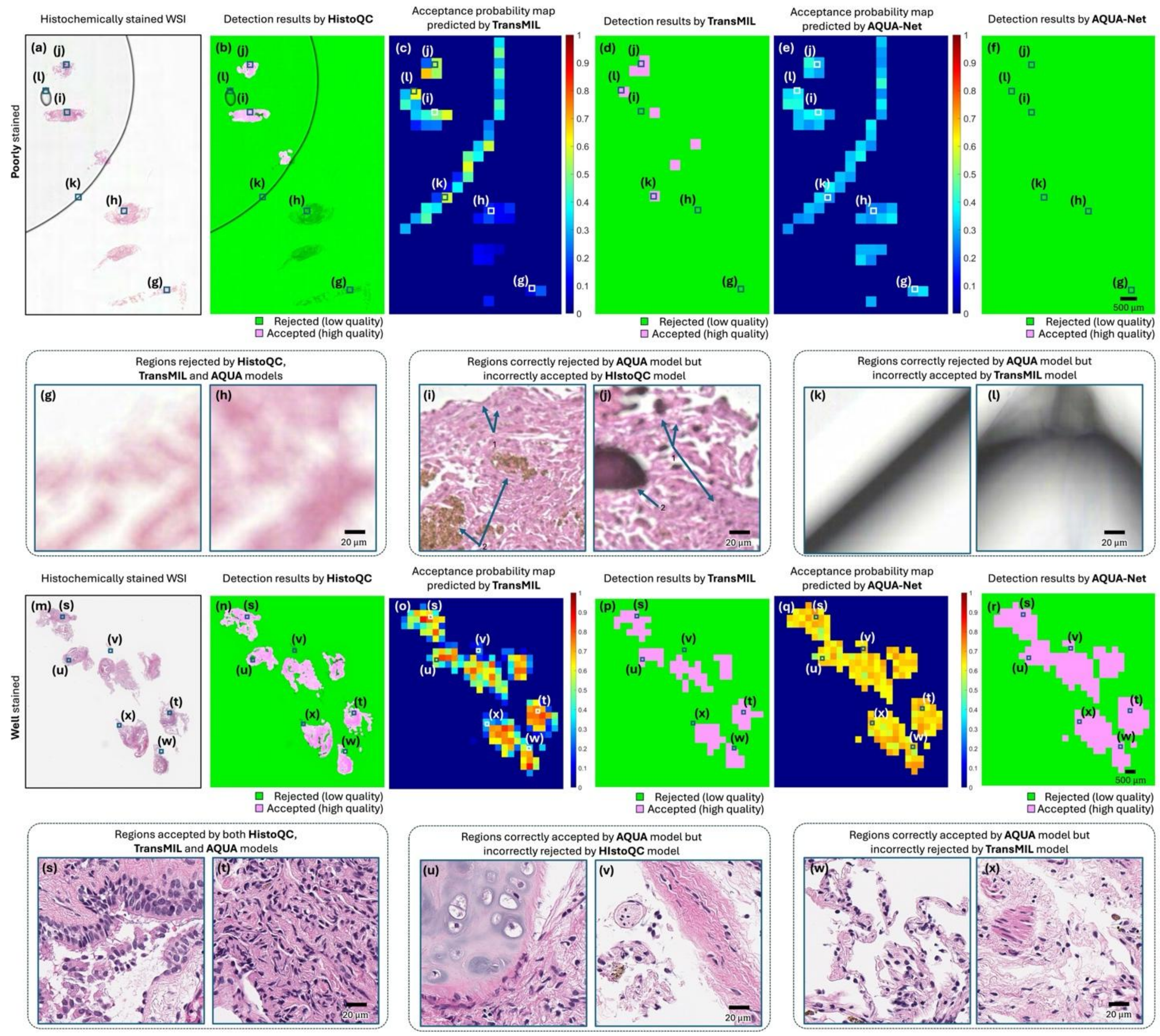

Supplementary Figure 3 Additional examples of the quality control detection results from the HistoQC model, the TransMIL model and the AQuA model. (a) The WSI of a poorly stained HS slide labelled by pathologists. (b) The detection results of (a) using the HistoQC model, where pink regions indicate high-quality stained areas accepted by the model, and green regions correspond to poorly stained areas rejected by the quality control model. (c) The acceptance probability map of (a) predicted by the TransMIL model, where values close to 1 indicate acceptance, while values near 0 signify rejection. (d) The detection results of (a) using the TransMIL model, which was obtained by thresholding (c) by 0.5. (e) The acceptance probability map of (a) predicted by the AQuA model. (f) The detection results of (a) using the AQuA model, which was obtained by thresholding (e) by 0.5. (g-h) Two examples of zoomed-in tissue regions rejected by the HistoQC model, the TransMIL model and AQuA model, which contain out-of-focus areas. (i-j) Two examples of zoomed-in regions with out-of-focus areas (arrows 1 and 2 in (i-j)), purple staining artifacts (arrow 2 in (i)) and brown exogenous pigments masking the tissue (arrow 2 in (j)), which were accepted by the HistoQC model but rejected by the AQuA model. (k-l) Two examples of zoomed-in tissue regions corresponding to air bubbles, which are correctly rejected by the AQuA model, but incorrectly accepted by the TransMIL model. (m) The WSI of a well-stained HS slide labelled by pathologists. (n) The detection results of (m) using the HistoQC model. (o) The acceptance probability map of (m) predicted by the TransMIL model. (p) The detection results of (m) using the TransMIL model, which was obtained by thresholding (o) by 0.5. (q) The acceptance probability map of (m) predicted by the AQuA model. (r) The detection results of (m) using the AQuA model, which was obtained by thresholding (q) by 0.5. (s) One example of a zoomed-in tissue region accepted by the HistoQC model, the TransMIL model and the AQuA model. (t) Another example of a zoomed-in tissue region accepted by the HistoQC model, the TransMIL model and the AQuA model. (u-v) Two examples of zoomed-in regions with good staining quality, which

were rejected by the HistoQC model but accepted by the AQuA model. (w-x) Another two examples of zoomed-in regions with good staining quality, which were rejected by the TransMIL model but accepted by the AQuA model.

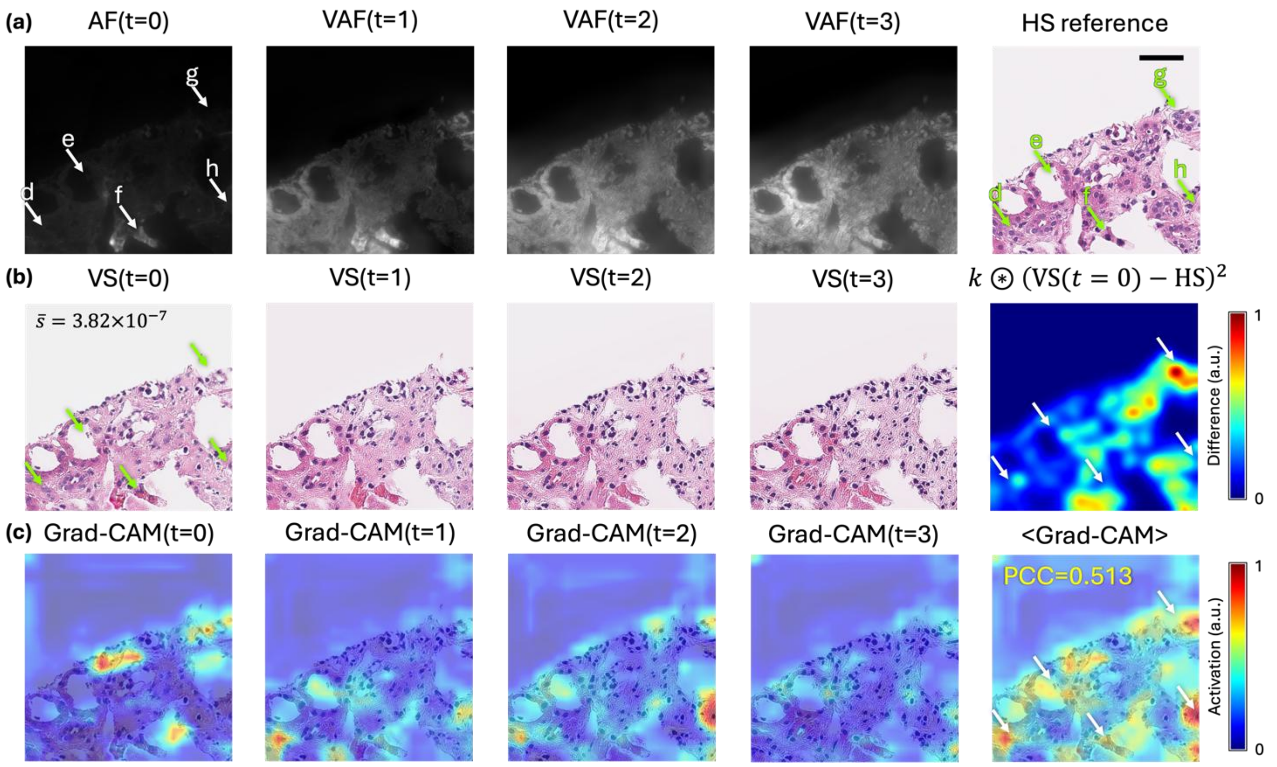

Supplementary Figure 4 Spatial-temporal Grad-CAM visualization of the activation maps during the AQuA inference on poor VS images of human kidney sample. (a) AF and VAF images in the VS-AF iterations, and the HS reference image (ground truth). (b) VS images in the VS-AF iterations, and the difference map between the VS(t=0) and the HS reference image is smoothed by a Gaussian kernel k with $\sigma = 15.5\text{px}$ to match the resolution of Grad-CAM. $\circledast$ stands for 2D convolution. (c) Grad-CAM heatmaps for VS(t) and the averaged heatmap. <·> stands for averaging over time. PCC values were calculated between the difference map and the averaged heatmap. (d) Nuclei artifacts generated by virtual staining were successfully identified by AQuA without relying on an HS reference image. (e) A tiny residual tissue not cleared in sample preparation was successfully detected by AQuA. (f) Significant artifacts and hallucinatory tissues generated by virtual staining were successfully detected by AQuA without relying on an HS reference image. (g) Aggregate of large nuclei identified by AQuA without relying on an HS reference image. (h) Hallucinatory, irregular nuclei detected by AQuA. (d, e, f, g, h) are pointed by arrows in (a). Scale bar: 50μm.

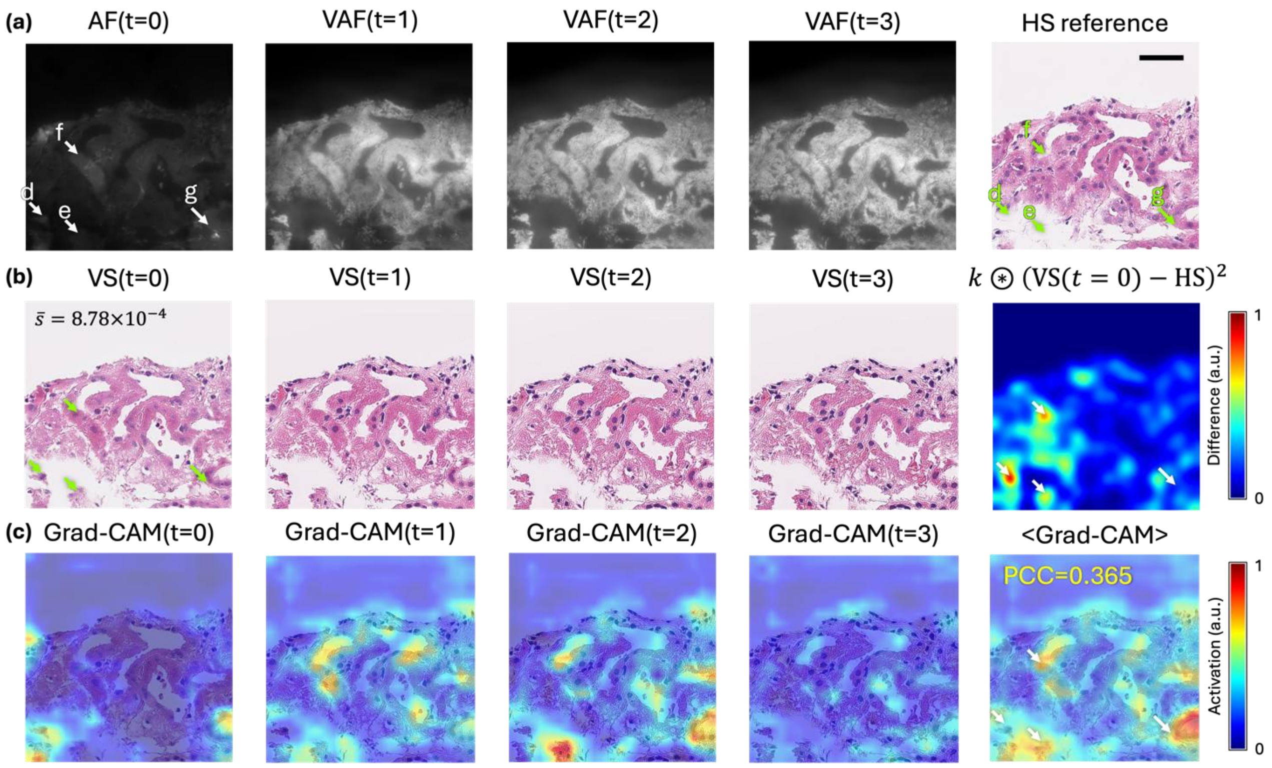

Supplementary Figure 5 Spatial-temporal Grad-CAM visualization of the activation maps during the AQuA inference on poor VS images of human kidney sample. (a) AF and VAF images in the VS-AF iterations, and the HS reference image (ground truth). (b) VS images in the VS-AF iterations, and the difference map between the VS(t=0) and the HS reference image is smoothed by a Gaussian kernel k with $\sigma = 15.5\text{px}$ to match the resolution of Grad-CAM. $\circledast$ stands for 2D convolution. (c) Grad-CAM heatmaps for VS(t) and the averaged heatmap. <·> stands for averaging over time. PCC values were calculated between the difference map and the averaged heatmap. (d, e) Small fragmented areas detected by AQuA without relying on an HS reference image. Note that parts of the tissue in the corresponding regions were not present in the HS reference. (f, g) Areas with high AF intensity not derived from tissue morphology were successfully detected by AQuA. The HS reference confirmed AQuA predictions. Scale bar: 50μm.

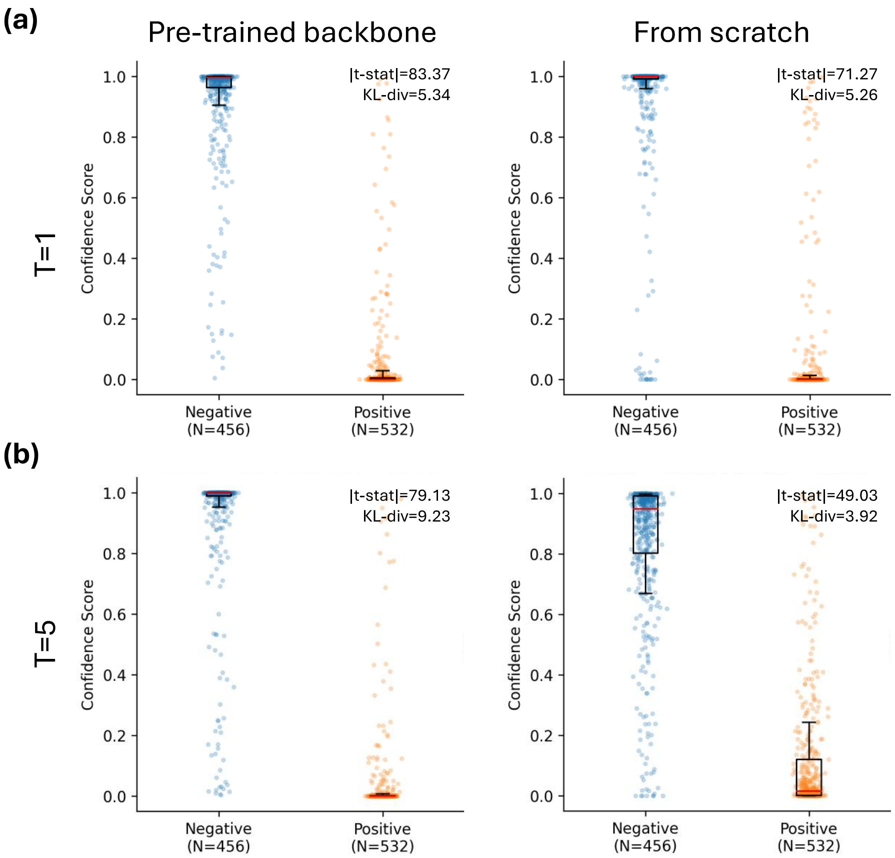

Supplementary Figure 6 An ablation study on the pre-trained ResNet-50 backbone. Predictions of two ResNet-50 classifiers with and without pre-trained backbone on the kidney VS image test set, (a) with T=1 and (b) with T=5. Both models were trained on the same training set for the same number of epochs. Box plot, 25th-75th percentiles; center, median; whisker, 16th-84th percentiles.

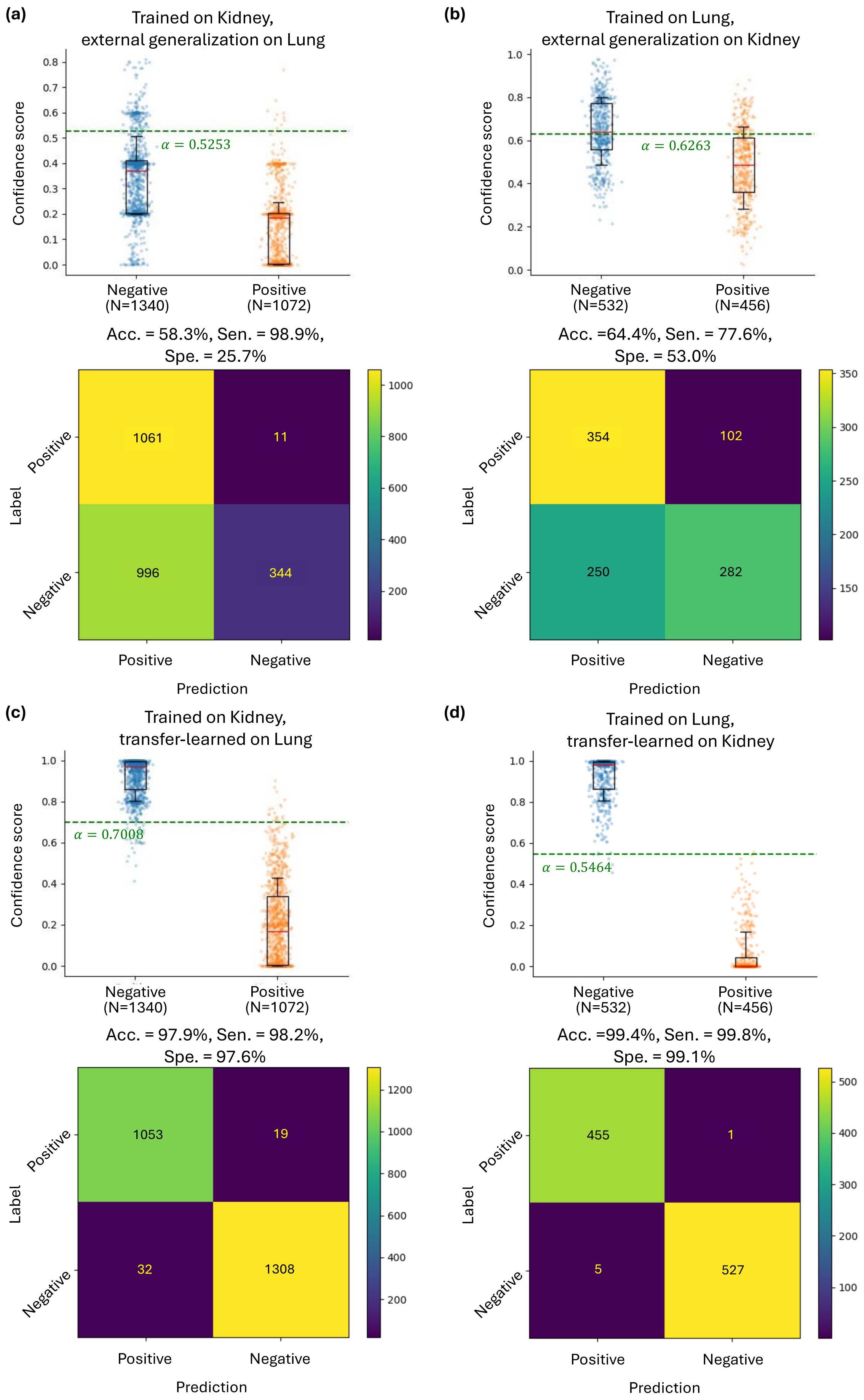

Supplementary Figure 7 External generalization of AQuA on unseen types of tissue samples. Classification

performance of (a) AQuA trained on human kidney samples and tested on human lung samples to test external generalization; (b) AQuA trained on human lung samples and tested on human kidney samples to test external generalization; (c) AQuA trained on human kidney samples, then transfer-learned and tested on human lung samples; (d) AQuA trained on human lung samples, then transfer-learned and tested on human kidney samples. Threshold α was selected as the 100% sensitivity threshold on the validation set. Box plot, 25th-75th percentiles; center, median; whisker, 16th-84th percentiles. Refer to the Methods section for transfer learning details.

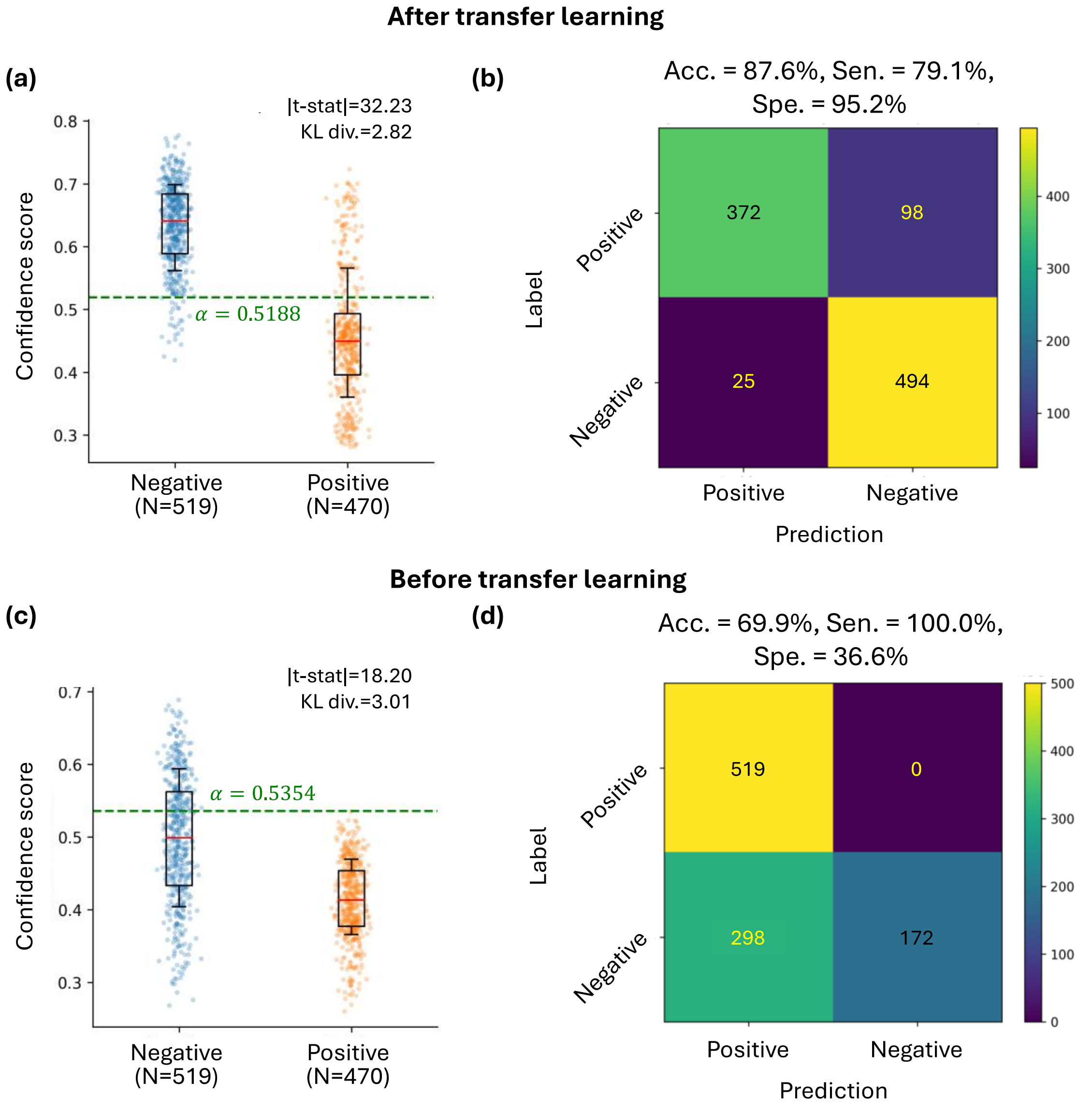

Supplementary Figure 8 Autonomous quality assessment of AQuA model before and after transfer learning on a subset of good HS images from the TCGA dataset. (a) Boxplot of confidence scores given by AQuA after transfer learning. (b) Confusion matrix of AQuA's testing result on TCGA dataset after transfer learning. (c) Same as (a), but using the AQuA model before transfer learning. (d) Same as (b), but using the AQuA model before transfer learning.

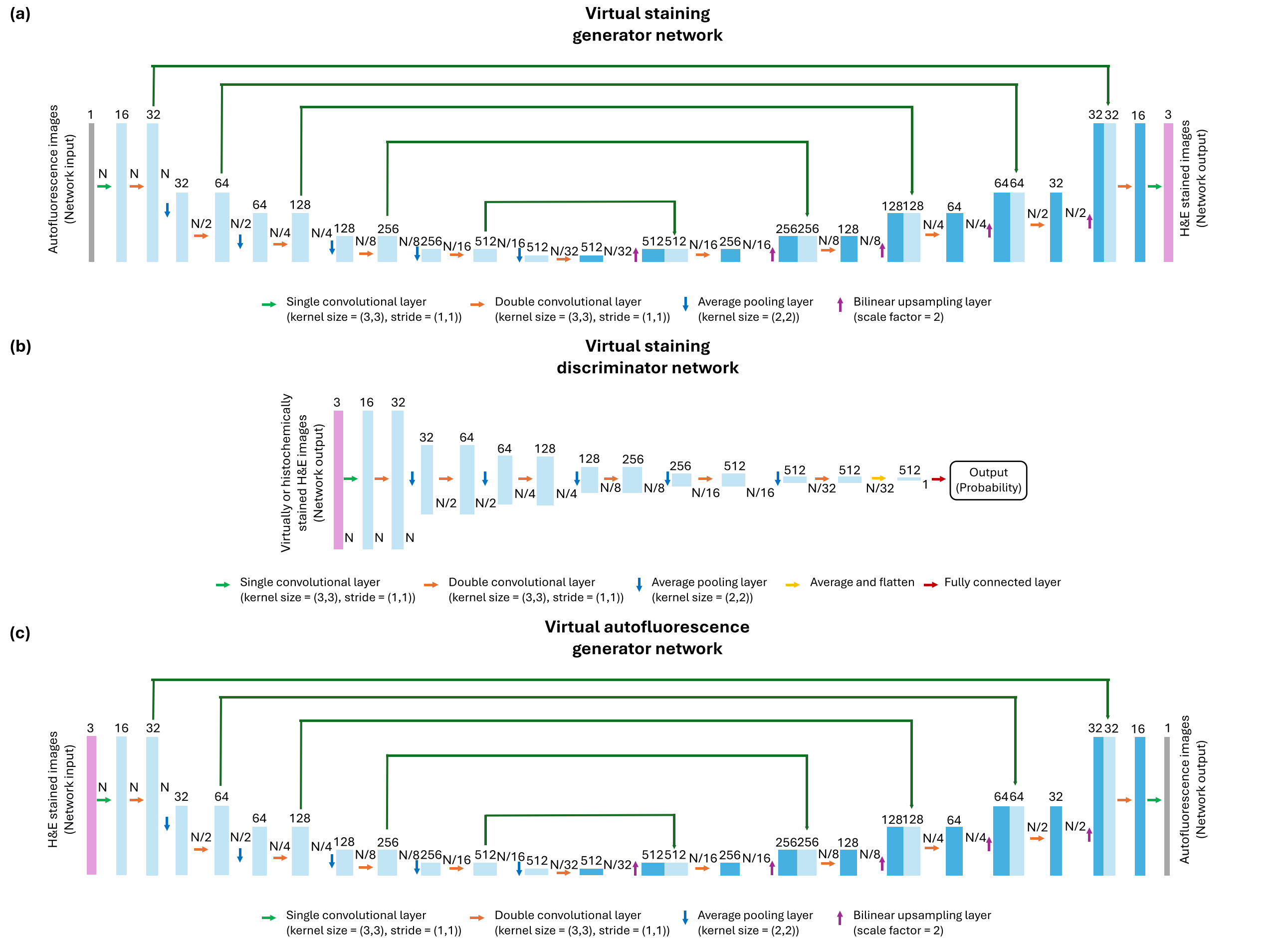

Supplementary Figure 9 Network architectures: (a) the virtual staining network, (b) the discriminator network and (c) the virtual autofluorescence network.

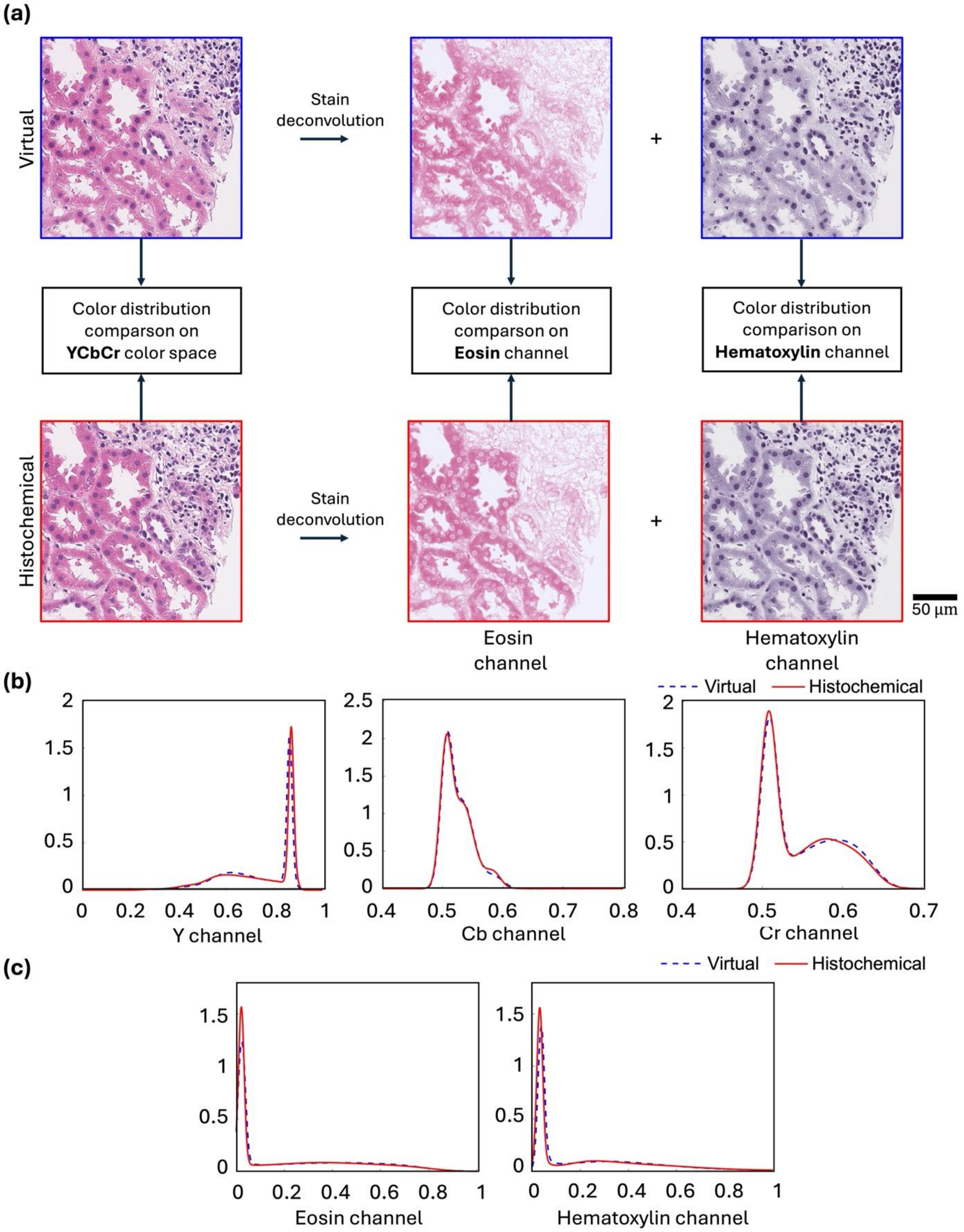

Supplementary Figure 10 Color distribution comparisons of paired VS and HS kidney tissue images. (a) the workflow of comparing color distributions of the VS and HS images in the YCbCr color space, the eosin channel and the hematoxylin channel. (b) The intensity histograms of the VS and HS images in the Y channel (left), Cb channel (middle), and Cr channel (right). (c) The intensity histograms of the VS and HS images in the eosin channel (left) and the hematoxylin channel (right).

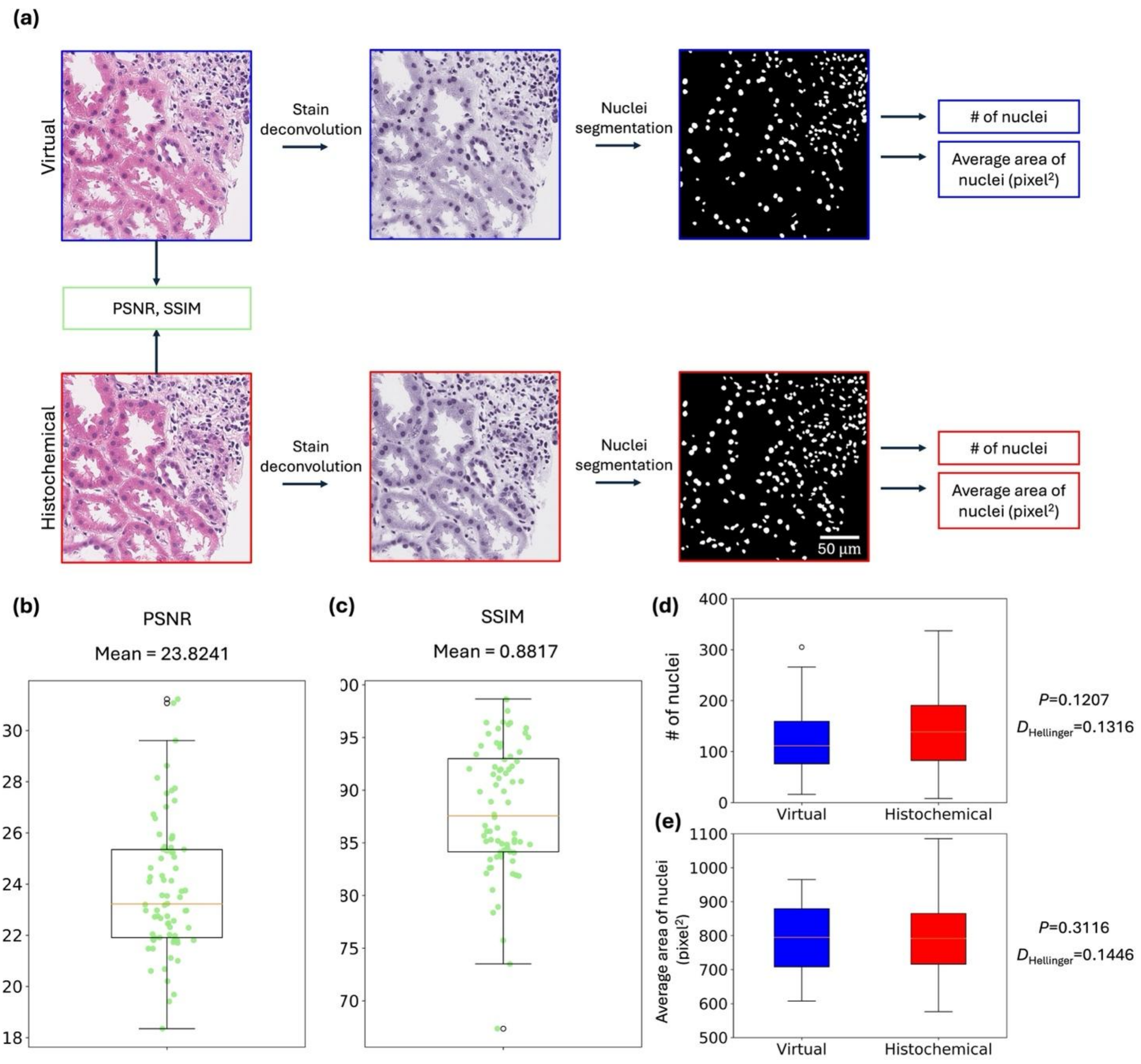

Supplementary Figure 11 PSNR, SSIM and structural feature comparisons of paired VS and HS kidney tissue images. (a) the workflow of comparing PSNR, SSIM, and different features (# of nuclei per FOV and average nuclei area) between the VS and HS images. (b) The box plot of PSNR when comparing all 76 test VS and HS image pairs. (c) The box plot of SSIM when comparing all 76 test VS and HS image pairs. (d) The box plots of the distributions of "# of nuclei per FOV" for the VS and HS images, with a Hellinger distance of 0.1316 and a *P* score of 0.1207, indicating a non-significant difference. (e) The box plots of the distributions of "average area of nuclei (pixel^2)" for the VS and HS images, with a Hellinger distance of 0.1446 and a *P* score of 0.3116, also indicating a non-significant difference. Box plot, 25th-75th percentiles; center, median; whisker, 0th-100th percentiles excluding outliers. (Refer to the Methods section for the implementation of the evaluation metrics and the statistical tests).

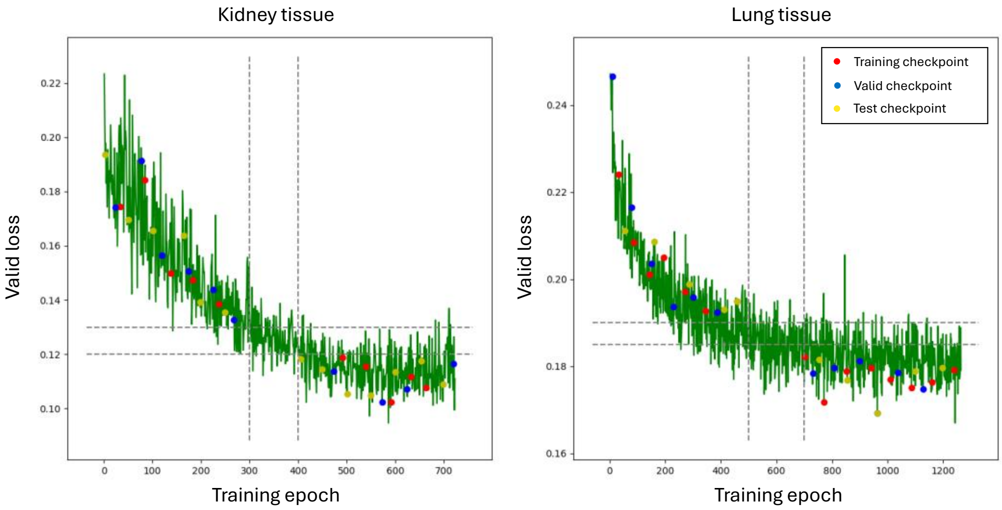

Supplementary Figure 12 Thresholding and selection of the VS network model checkpoints. The gray zones exclude checkpoints with ambiguity so that the selected checkpoints are well separated in both the validation loss and training epoch number. Refer to the Methods section for more details on thresholding. The legends denote three types of VS checkpoints selected for the training, validation and testing of AQuA.
