# Supplementary material for: A robust and scalable framework for hallucination detection in virtual tissue staining and digital pathology
Source: Nat Biomed Eng. 2025 Jun 16;9(12):2196–214. doi: 10.1038/s41551-025-01421-9 (PMC12705451; doi:10.1038/s41551-025-01421-9)
Supplement: Supplementary file 2 — Reporting Summary [file 41551_2025_1421_MOESM2_ESM.pdf]

nature portfolio

Corresponding author(s): Aydogan Ozcan

Last updated by author(s): Apr 3, 2025

Reporting Summary

Nature Portfolio wishes to improve the reproducibility of the work that we publish. This form provides structure for consistency and transparency in reporting. For further information on Nature Portfolio policies, see our Editorial Policies and the Editorial Policy Checklist.

Statistics

For all statistical analyses, confirm that the following items are present in the figure legend, table legend, main text, or Methods section.

n/a	Confirmed	
☐	☒	The exact sample size (*n*) for each experimental group/condition, given as a discrete number and unit of measurement
☐	☒	A statement on whether measurements were taken from distinct samples or whether the same sample was measured repeatedly
☐	☒	The statistical test(s) used AND whether they are one- or two-sided<br>*Only common tests should be described solely by name; describe more complex techniques in the Methods section.*
☒	☐	A description of all covariates tested
☒	☐	A description of any assumptions or corrections, such as tests of normality and adjustment for multiple comparisons
☐	☒	A full description of the statistical parameters including central tendency (e.g. means) or other basic estimates (e.g. regression coefficient) AND variation (e.g. standard deviation) or associated estimates of uncertainty (e.g. confidence intervals)
☐	☒	For null hypothesis testing, the test statistic (e.g. *F*, *t*, *r*) with confidence intervals, effect sizes, degrees of freedom and *P* value noted<br>*Give P values as exact values whenever suitable.*
☒	☐	For Bayesian analysis, information on the choice of priors and Markov chain Monte Carlo settings
☒	☐	For hierarchical and complex designs, identification of the appropriate level for tests and full reporting of outcomes
☒	☐	Estimates of effect sizes (e.g. Cohen's *d*, Pearson's *r*), indicating how they were calculated

Our web collection on statistics for biologists contains articles on many of the points above.

Software and code

Policy information about availability of computer code

Data collection	AF (autofluorescence) images of unlabeled kidney tissue slides were captured by an Olympus IX-83 microscope (controlled by MetaMorph microscope automation software) with a40 × /0.95NA (UPLSAPO, Olympus) objective lens, under the DAPI filter cube (Semrock OSFI3-DAPI5060C, EX 377/50 nm EM 447/60 nm). Similarly, for unlabeled lung tissue slides, the AF images were obtained using a Leica DMI8 microscope (controlled by Leica LAS X microscopy automation software) with a 40×/0.95 NA objective lens (Leica HC PL APO 40×/0.95 DRY), under the DAPI filter cube. After undergoing standard histochemical H&E staining, the stained tissue slides were digitized using a Leica Biosystems Aperio AT2 brightfield slide scanner.
Data analysis	The codes for the deep learning models used in this work (written in Python 3.9.16 and PyTorch 1.13.0) can be accessed through: https://drive.google.com/drive/folders/1ztfS6hTkyU-mUrXuHI6LAgUFJ1-hcEyY?usp=share_link<br>Upon acceptance of the manuscript, it will also be placed in GitHub for public release. The trained model and demo data are uploaded and available in the same code repository. The code for analyzing the results was written in Python using standard, open-source Python libraries.

For manuscripts utilizing custom algorithms or software that are central to the research but not yet described in published literature, software must be made available to editors and reviewers. We strongly encourage code deposition in a community repository (e.g. GitHub). See the Nature Portfolio guidelines for submitting code & software for further information.

Data

Policy information about availability of data

All manuscripts must include a data availability statement. This statement should provide the following information, where applicable:

- Accession codes, unique identifiers, or web links for publicly available datasets
- A description of any restrictions on data availability
- For clinical datasets or third party data, please ensure that the statement adheres to our policy

A portion of the testing dataset is also shared and referenced in the code repository without any links or identifiers to the patients, which is made available at: https://drive.google.com/drive/folders/1ztfS6hTkyU-mUrXuHI6LAgUFJ1-hcEyY?usp=share_link. The TCGA dataset (an open-sourced and publicly available dataset) labels on human lung tissue WSIs are also shared in the same repository.

Research involving human participants, their data, or biological material

Policy information about studies with human participants or human data. See also policy information about sex, gender (identity/presentation), and sexual orientation and race, ethnicity and racism.

Reporting on sex and gender

Use the terms sex (biological attribute) and gender (shaped by social and cultural circumstances) carefully in order to avoid confusing both terms. Indicate if findings apply to only one sex or gender; describe whether sex and gender were considered in study design; whether sex and/or gender was determined based on self-reporting or assigned and methods used. Provide in the source data disaggregated sex and gender data, where this information has been collected, and if consent has been obtained for sharing of individual-level data; provide overall numbers in this Reporting Summary. Please state if this information has not been collected. Report sex- and gender-based analyses where performed, justify reasons for lack of sex- and gender-based analysis.

Reporting on race, ethnicity, or other socially relevant groupings

Please specify the socially constructed or socially relevant categorization variable(s) used in your manuscript and explain why they were used. Please note that such variables should not be used as proxies for other socially constructed/relevant variables (for example, race or ethnicity should not be used as a proxy for socioeconomic status). Provide clear definitions of the relevant terms used, how they were provided (by the participants/respondents, the researchers, or third parties), and the method(s) used to classify people into the different categories (e.g. self-report, census or administrative data, social media data, etc.) Please provide details about how you controlled for confounding variables in your analyses.

Population characteristics

Describe the covariate-relevant population characteristics of the human research participants (e.g. age, genotypic information, past and current diagnosis and treatment categories). If you filled out the behavioural & social sciences study design questions and have nothing to add here, write "See above."

Recruitment

Describe how participants were recruited. Outline any potential self-selection bias or other biases that may be present and how these are likely to impact results.

Ethics oversight

Identify the organization(s) that approved the study protocol.

Note that full information on the approval of the study protocol must also be provided in the manuscript.

Field-specific reporting

Please select the one below that is the best fit for your research. If you are not sure, read the appropriate sections before making your selection.

☒ Life sciences ☐ Behavioural & social sciences ☐ Ecological, evolutionary & environmental sciences

For a reference copy of the document with all sections, see nature.com/documents/nr-reporting-summary-flat.pdf

Life sciences study design

All studies must disclose on these points even when the disclosure is negative.

Sample size

After data preprocessing, we obtained 1054 non-overlapping AF-H&E image patch pairs (1424×1424 pixels) for kidney samples, and 1068 pairs (1024×1024 pixels) for lung samples, to train and validate the VS and VAF networks. The kidney samples originated from 10 unique patients (7 used for training and validating the VS/VAF and AQuA models, and 3 for testing), while the lung samples came from 29 unique patients. Among these 29 unique patients, 18 cases were allocated for training and validating the VS/VAF models, 7 out of these 18 cases were chosen for training and validating the AQuA model. The rest of the 11 cases were left for testing the AQuA model. For AQuA on human kidney samples, a set of 1054 non-overlapping AF FOVs (1424×1424 pixels) were collected from 7 individuals for training and validation, and a set of 76 non-overlapping AF FOVs collected from another 3 subjects were used for testing. For AQuA on human lung samples, we collected 572 non-overlapping AF FOVs (1024×1024 pixels) from 7 individuals for training and validation (a subset of the training and validation data of VS models), and 268 non-overlapping AF FOVs from another subject for testing. Additionally, the histochemical-stained H&E image dataset of human lung tissue sections consists of a subset of 100 non-overlapping FOVs (2048×2048 pixels) for training, 16 non-overlapping FOVs for validation, and 220 non-overlapping FOVs for testing of AQuA models. Moreover, for the external generalization test using TCGA dataset, two small subsets including 36 patches from 8 good whole slide images (WSIs), and 8 patches from 2 good WSIs were used as the negative

	samples for training and validation, respectively – i.e., no bad WSIs were used in the transfer learning step. The rest of the 989 patches (519 good and 470 bad patches) generated from 385 good WSIs and 66 bad WSIs were used for blind testing.
Data exclusions	The training FOVs and testing FOVs were exclusive, and the testing FOVs were strictly different from the training slides.
Replication	VS and VAF models were trained on two datasets of human kidney and lung tissue samples, and then blindly tested on corresponding test sets. 10 ensembles of AQuA model were repeated for each training dataset and combination hyperparameters. After training, each ensemble was blindly tested on the corresponding test set.
Randomization	The training, validation and testing image datasets were randomly partitioned. Ensembles used randomly generated seeds and initializations.
Blinding	All the testing results generated by the trained neural networks were blindly performed on new FOVs excluded from the training dataset. The blind testing FOVs were also captured on new slides from new patients that did not appear in the training dataset.

Reporting for specific materials, systems and methods

We require information from authors about some types of materials, experimental systems and methods used in many studies. Here, indicate whether each material, system or method listed is relevant to your study. If you are not sure if a list item applies to your research, read the appropriate section before selecting a response.

Materials & experimental systems

n/a	Involved in the study	
☒	☐	Antibodies
☒	☐	Eukaryotic cell lines
☒	☐	Palaeontology and archaeology
☒	☐	Animals and other organisms
☒	☐	Clinical data
☒	☐	Dual use research of concern
☒	☐	Plants

Methods

n/a	Involved in the study	
☒	☐	ChIP-seq
☒	☐	Flow cytometry
☒	☐	MRI-based neuroimaging

Plants

Seed stocks	*Report on the source of all seed stocks or other plant material used. If applicable, state the seed stock centre and catalogue number. If plant specimens were collected from the field, describe the collection location, date and sampling procedures.*
Novel plant genotypes	*Describe the methods by which all novel plant genotypes were produced. This includes those generated by transgenic approaches, gene editing, chemical/radiation-based mutagenesis and hybridization. For transgenic lines, describe the transformation method, the number of independent lines analyzed and the generation upon which experiments were performed. For gene-edited lines, describe the editor used, the endogenous sequence targeted for editing, the targeting guide RNA sequence (if applicable) and how the editor was applied.*
Authentication	*Describe any authentication procedures for each seed stock used or novel genotype generated. Describe any experiments used to assess the effect of a mutation and, where applicable, how potential secondary effects (e.g. second site T-DNA insertions, mosiacism, off-target gene editing) were examined.*
